# Supplementary material for: Genetic Diversity and Phenotypic Variation of Indigenous Wild Cherry Species in Kazakhstan and Uzbekistan
Source: Plants (Basel). 2025 May 30;14(11):1676. doi: 10.3390/plants14111676 (PMC12157008; doi:10.3390/plants14111676)
Supplement: Supplementary file 1 [file plants-14-01676-s001.zip › Supplementary material/Figure S1/Figure S1.pdf]

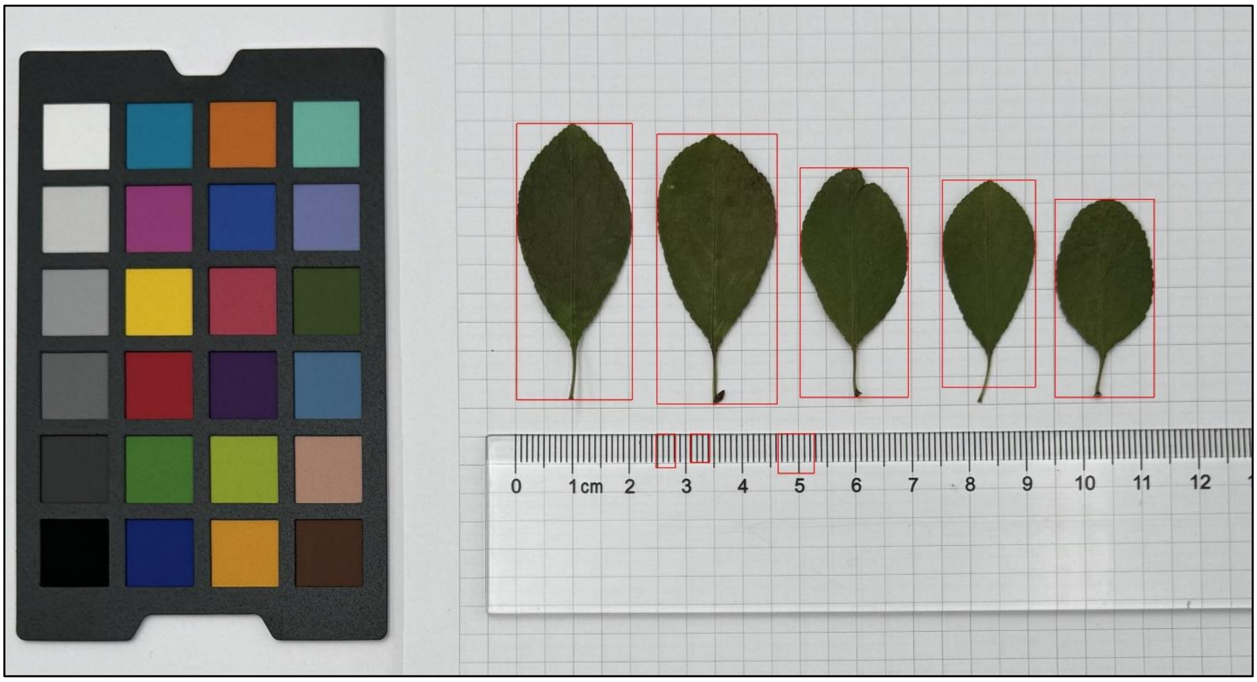

Figure S1. Prun24_069 *Prunus fruticosa*

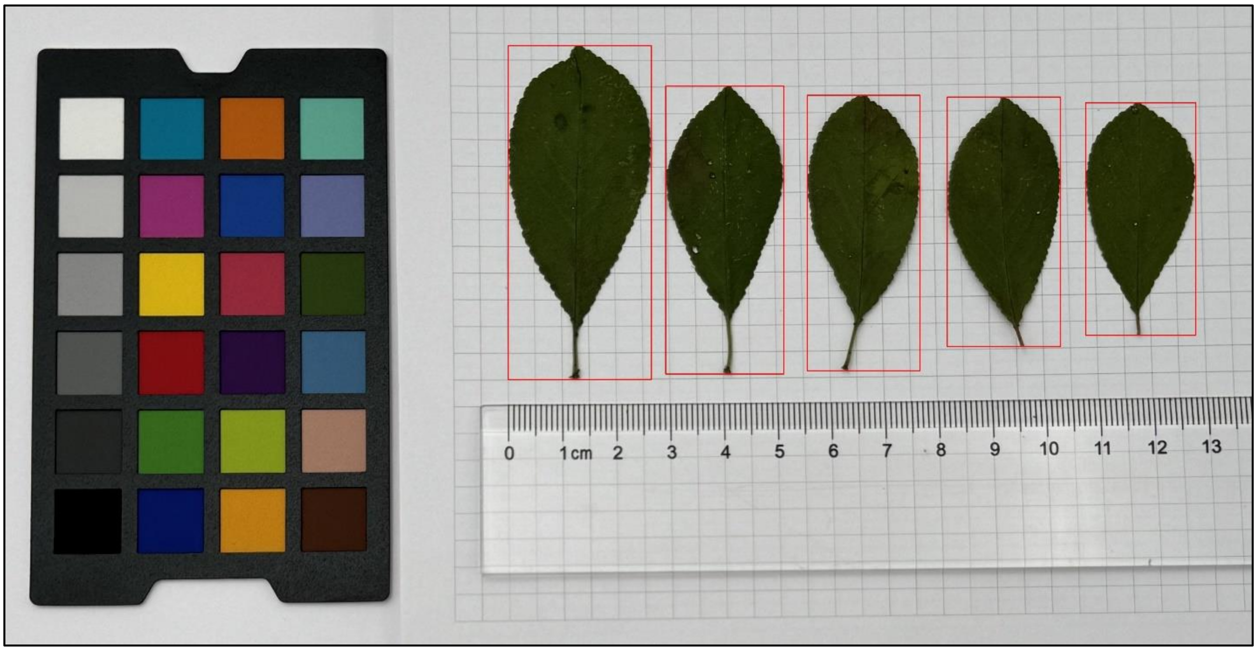

Figure S1. Prun24_075 *Prunus fruticosa*

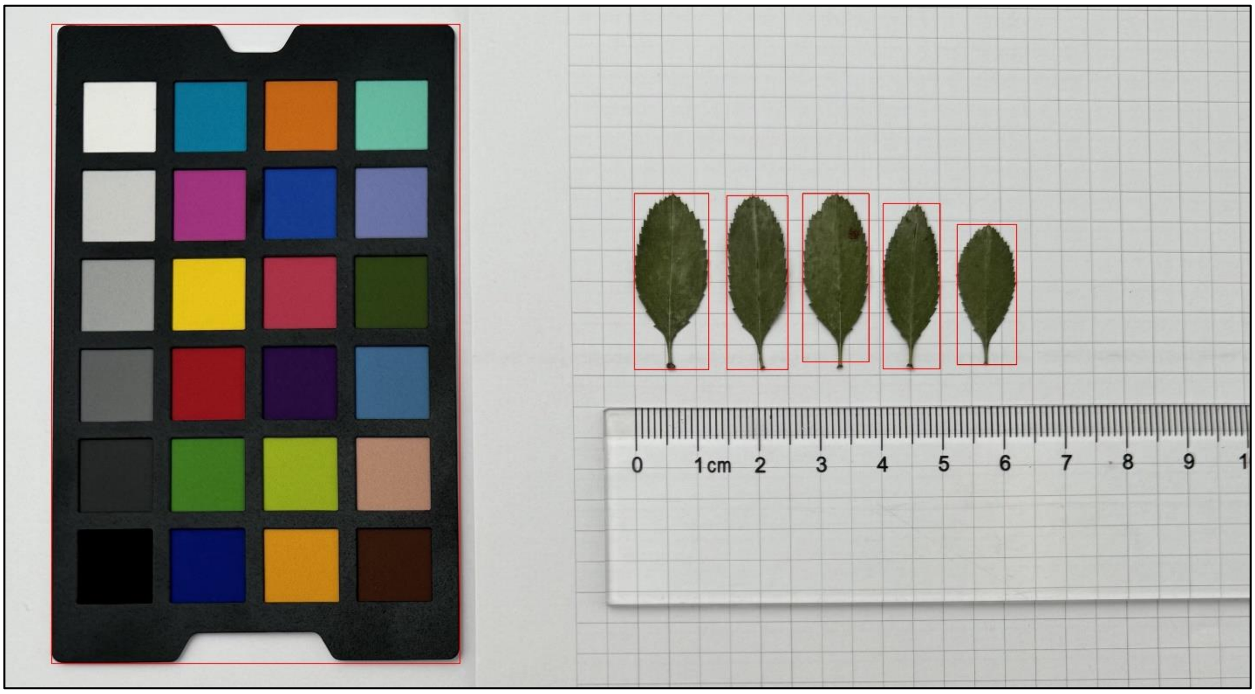

Figure S1. Prun24_108 *Prunus fruticosa*

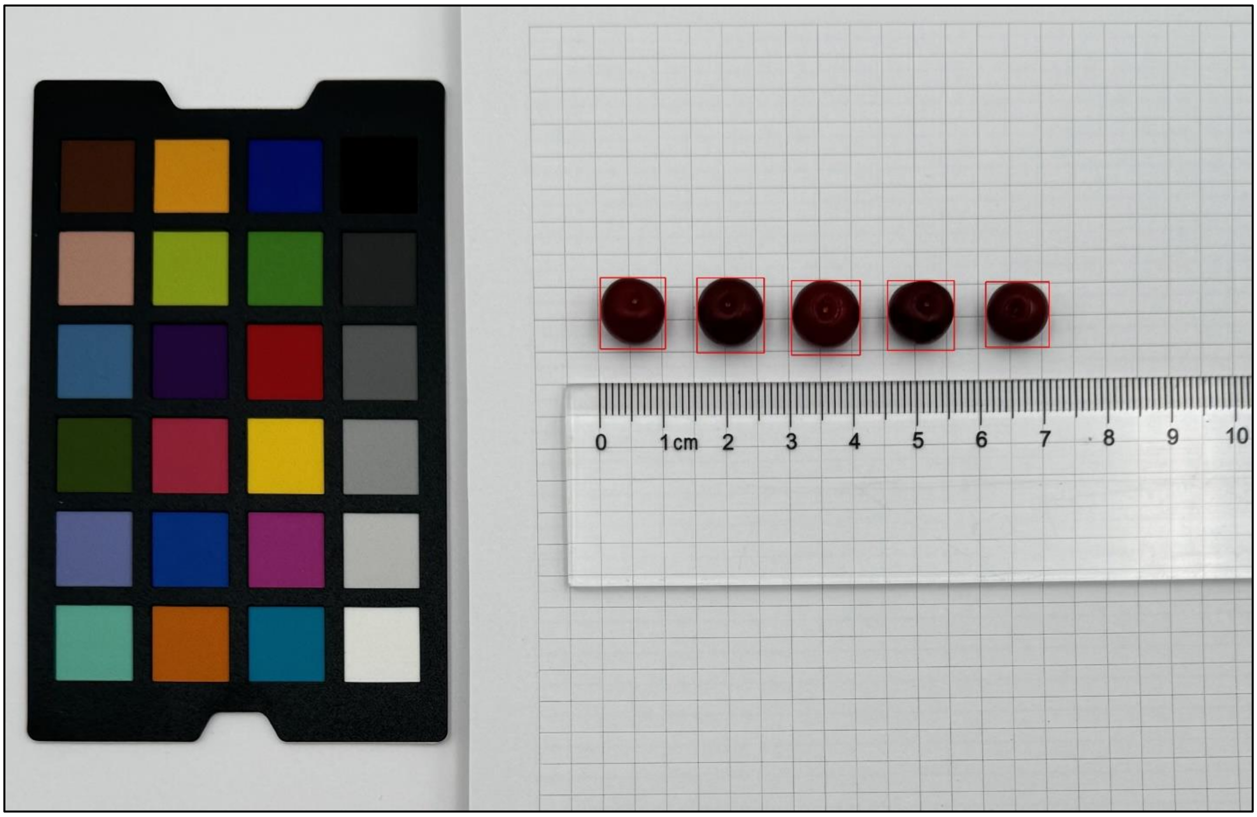

Figure S1. Prun24_130 *Prunus griifithi* var. *tianshanica*

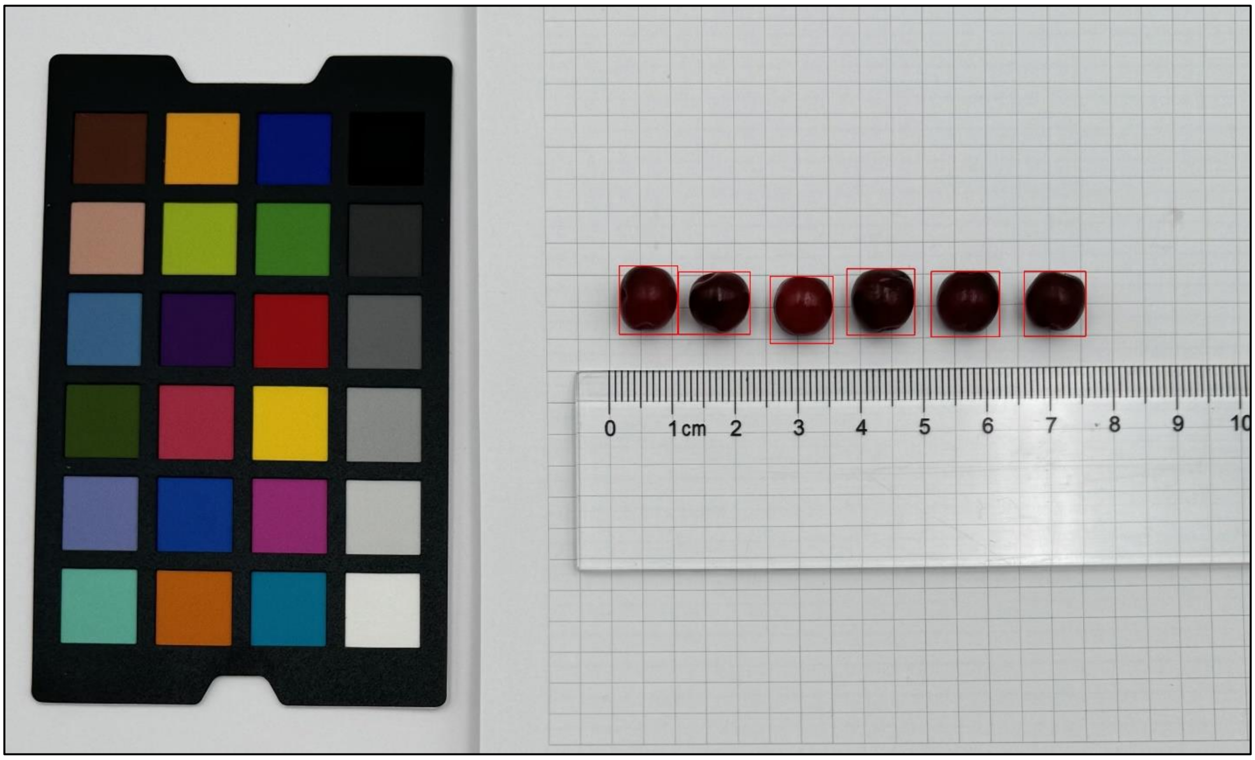

Figure S1. Prun24_132 *Prunus griifithi* var. *tianshanica*

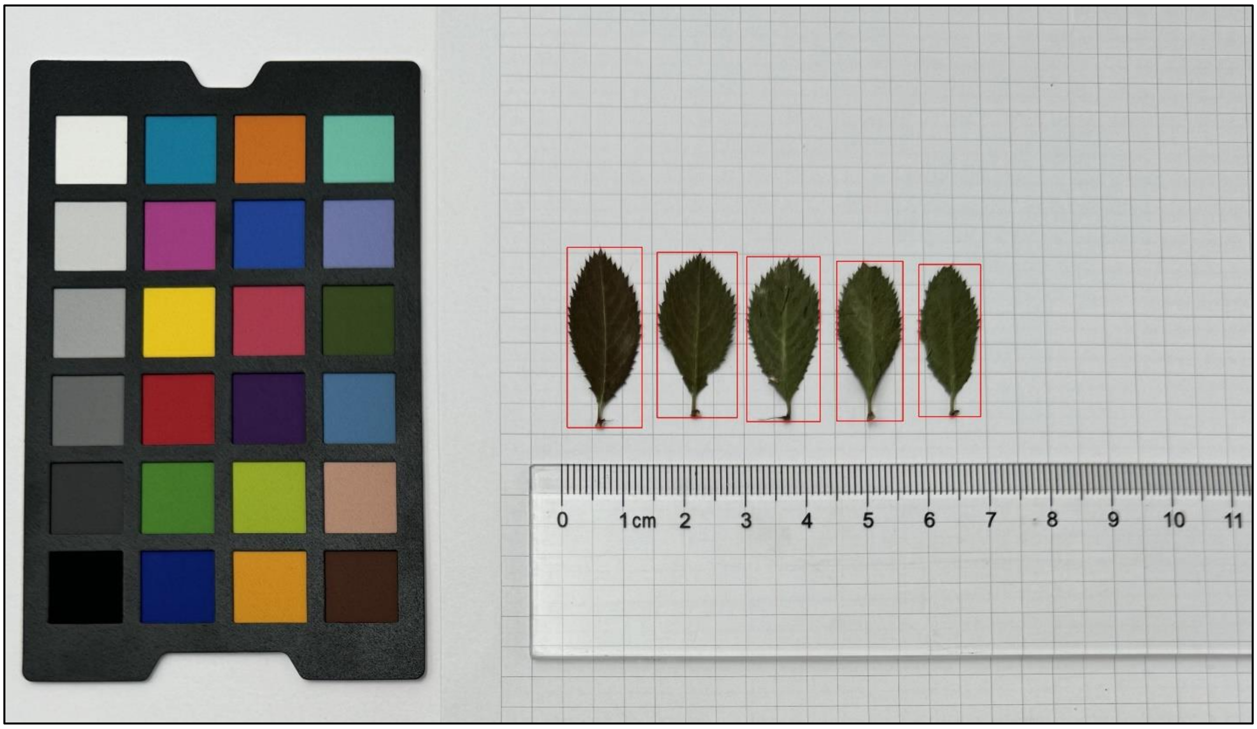

Figure S1. Prun24_135 *Prunus erythrocarpa*

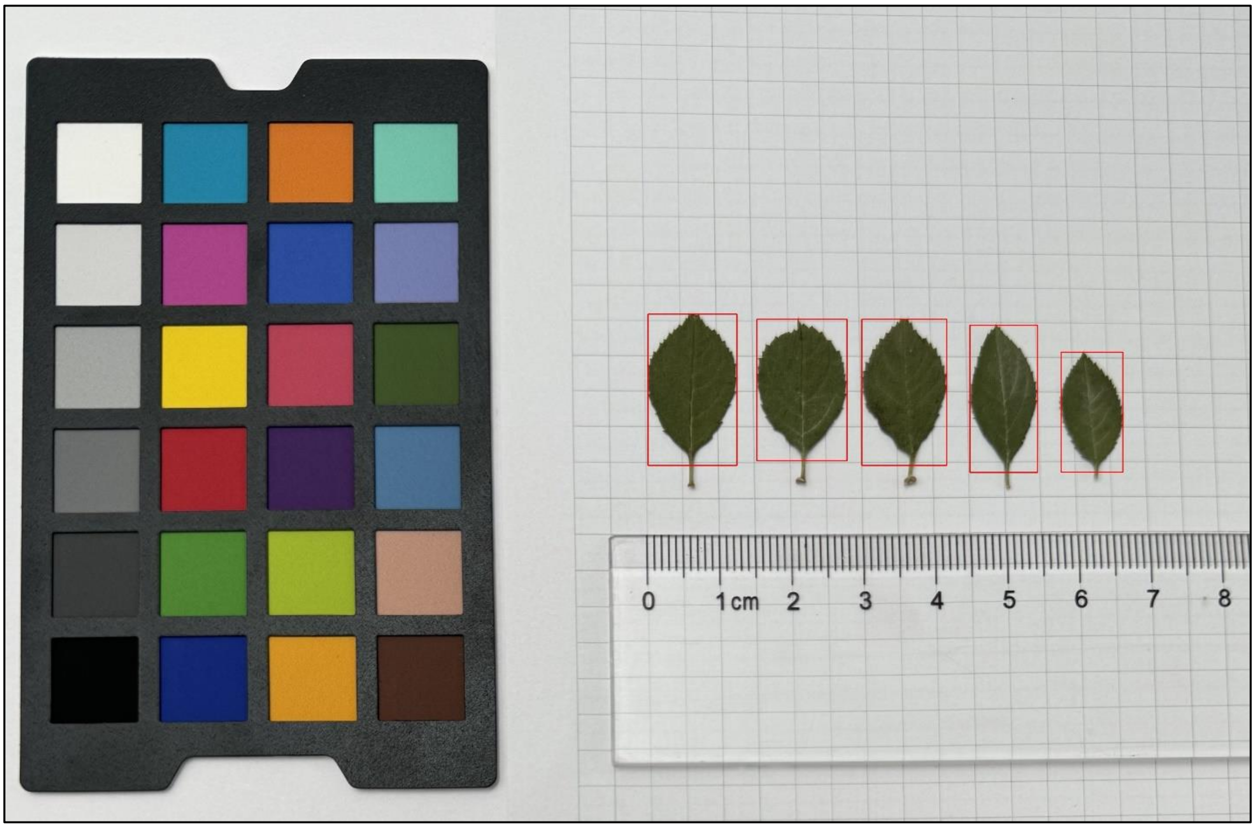

Figure S1. Prun24_136 *Prunus erythrocarpa*

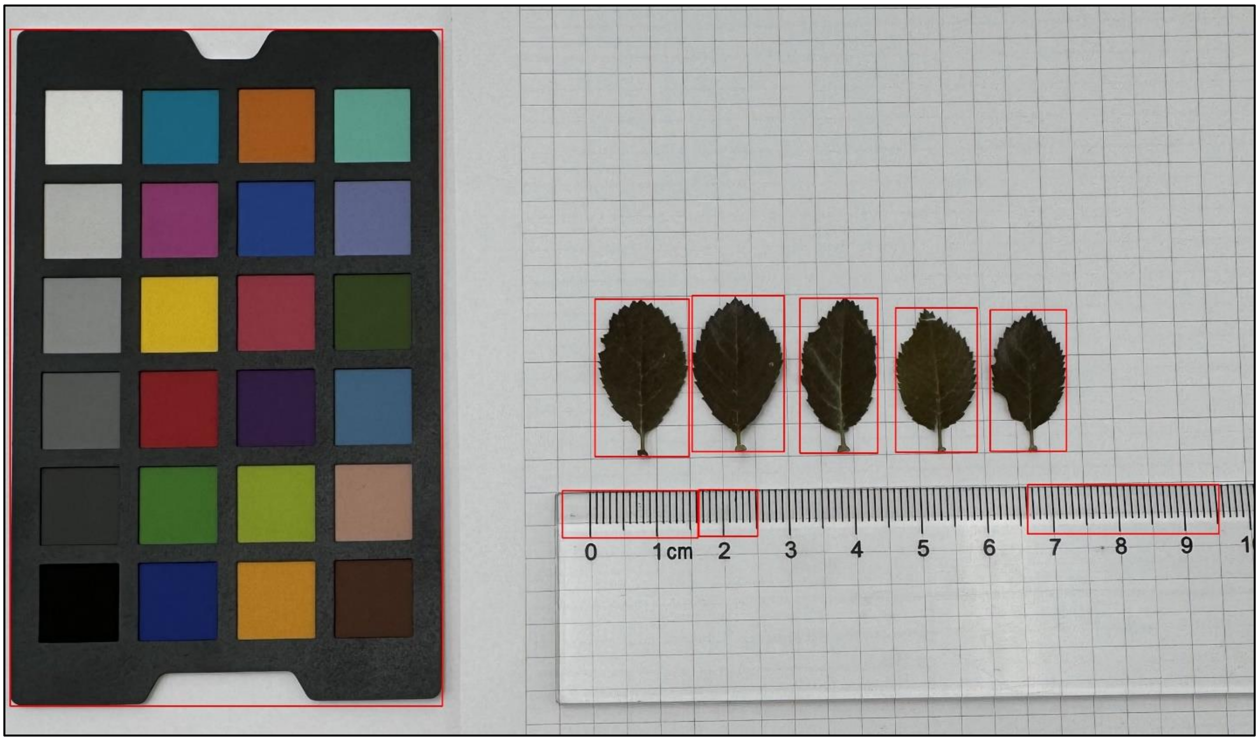

Figure S1. Prun24_141 *Prunus griifithi* var. *tianshanica*
