# Supplementary material for: Genetic Diversity and Phenotypic Variation of Indigenous Wild Cherry Species in Kazakhstan and Uzbekistan
Source: Plants (Basel). 2025 May 30;14(11):1676. doi: 10.3390/plants14111676 (PMC12157008; doi:10.3390/plants14111676)
Supplement: Supplementary file 1 [file plants-14-01676-s001.zip › Supplementary material/Figure S2/Figure S2.pdf]

Figure S2: Some images output from the 3730XL DNA Analyzer.

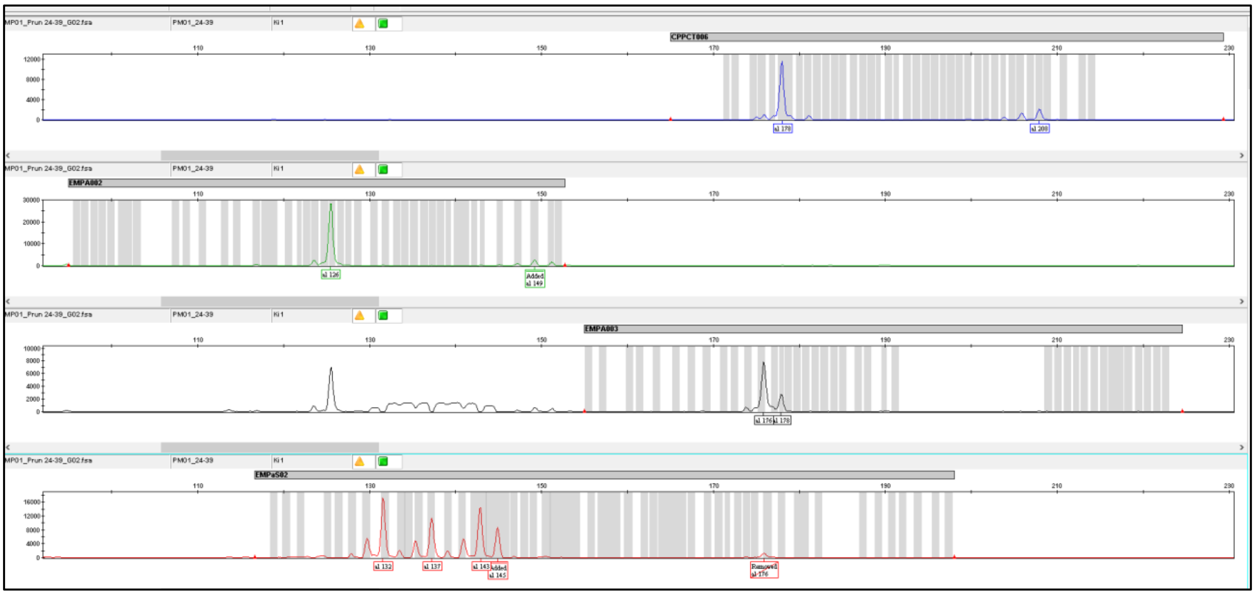

Primer Mix 01. Accessions: Prun24_039. *Prunus verrucosa*

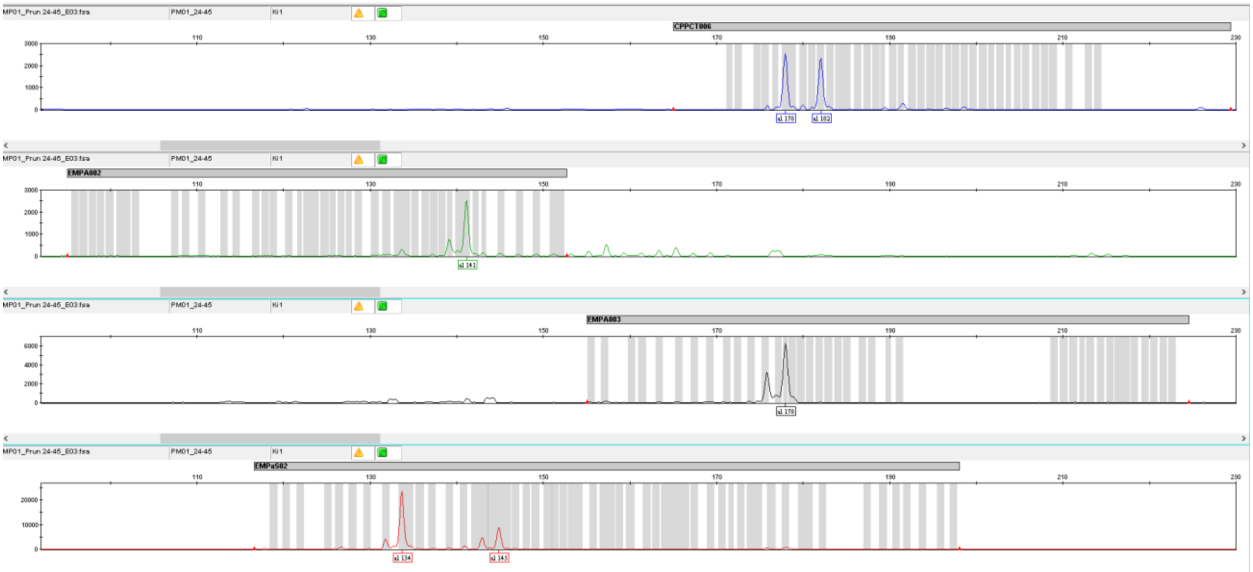

Primer Mix 01. Accessions: Prun24_045. *Prunus verrucosa*

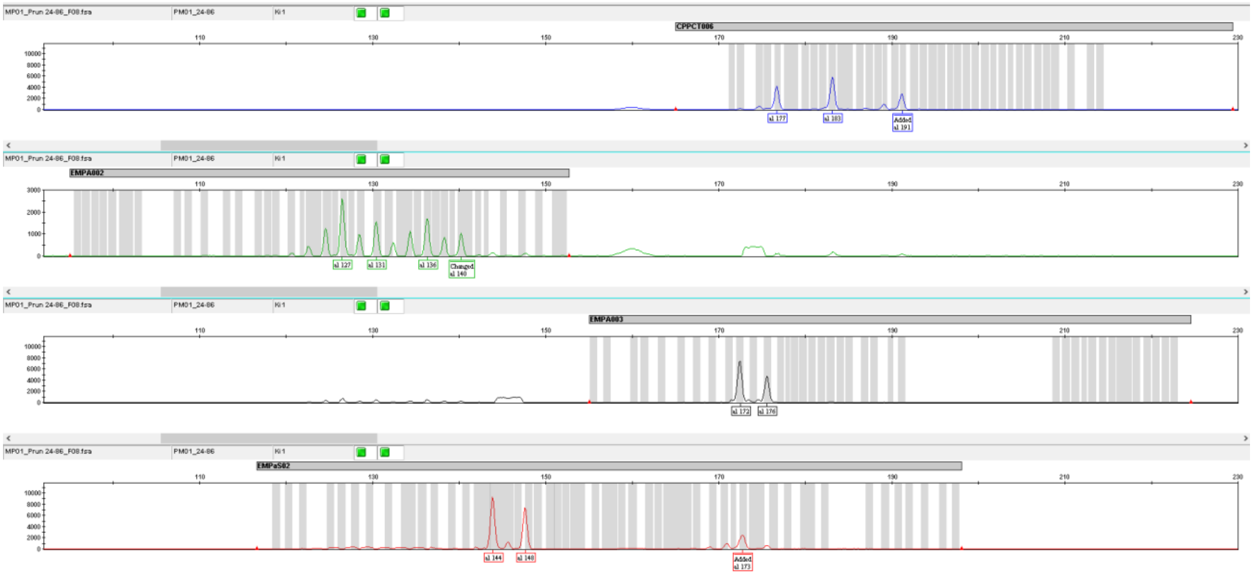

Primer Mix 01. Accessions: Prun24_086. *Prunus fruticosa*

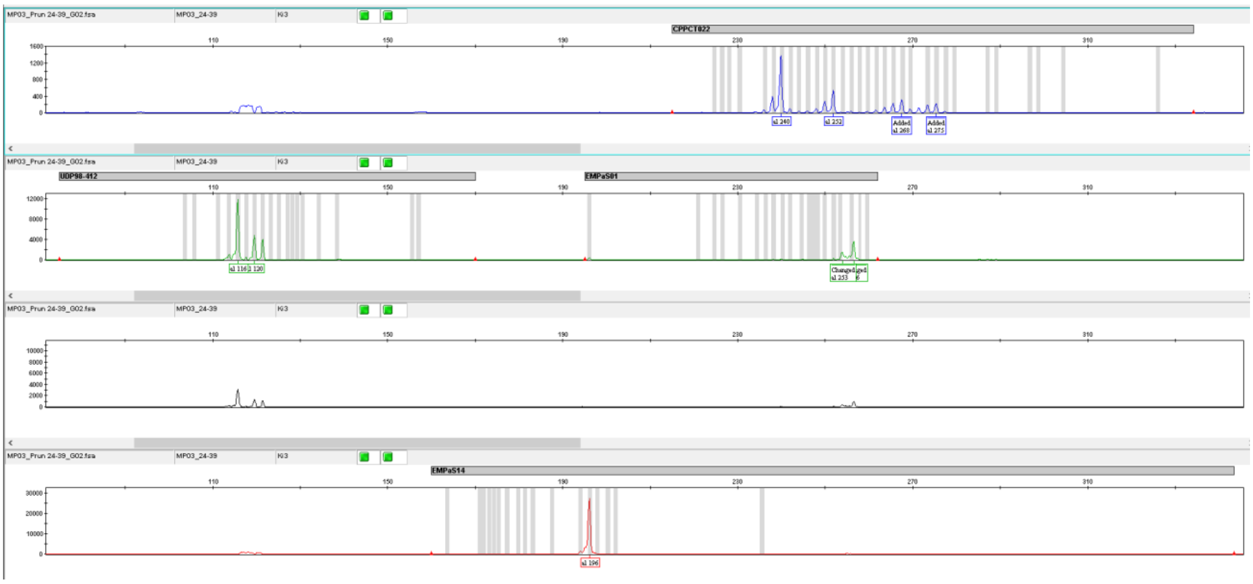

Primer Mix 02. Accessions: Prun24_039. *Prunus verrucosa*

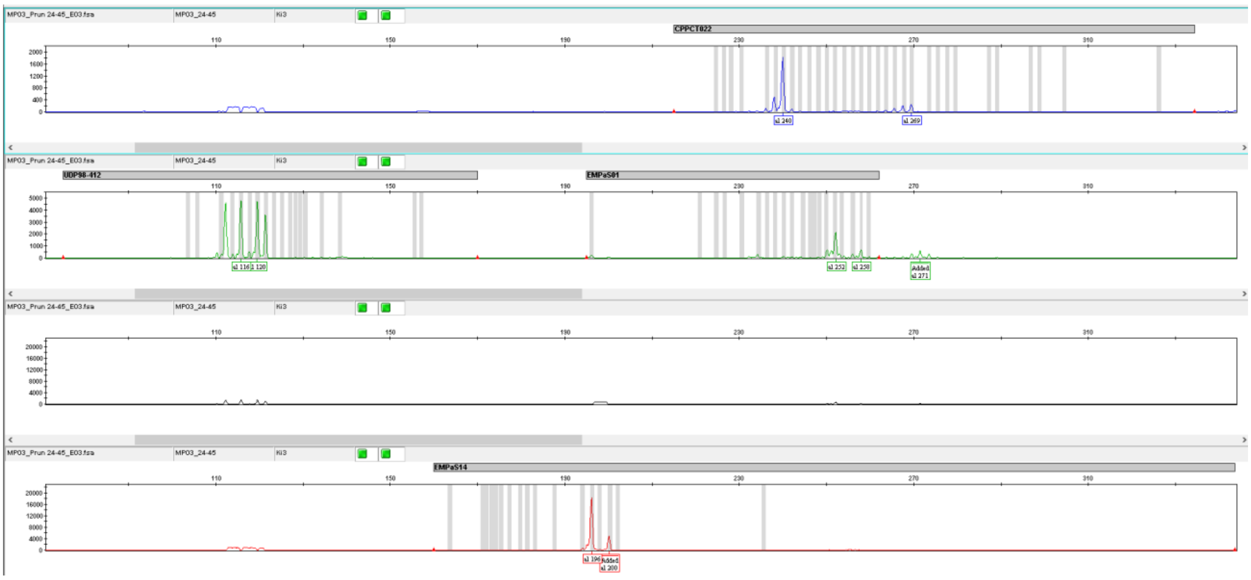

Primer Mix 02. Accessions: Prun24_039. *Prunus verrucosa*

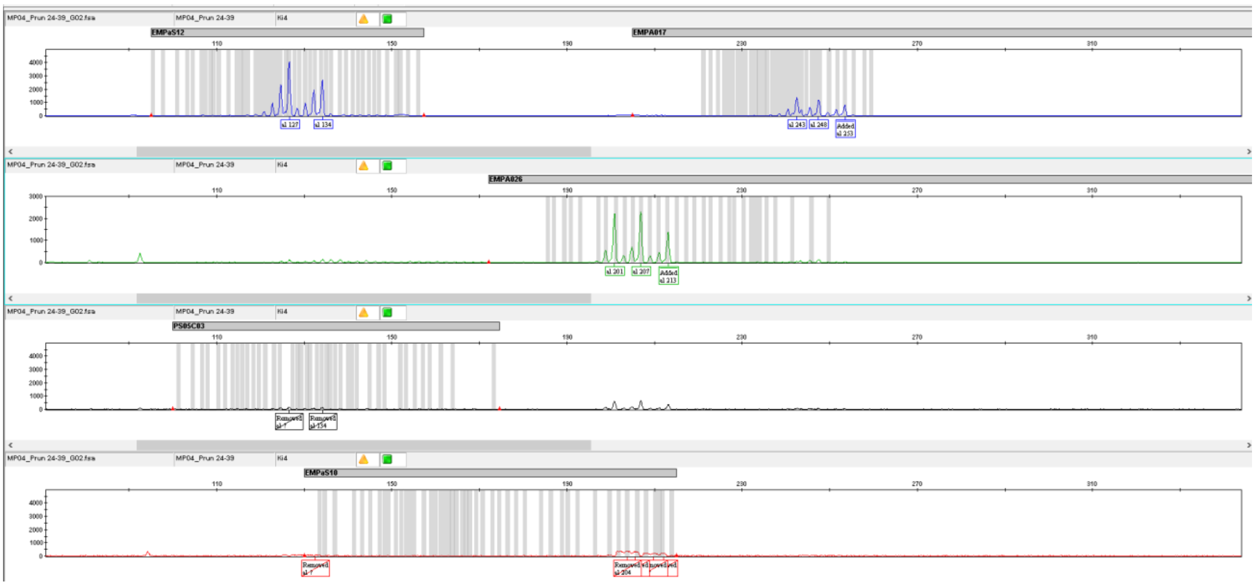

Primer Mix 03. Accessions: Prun24_039. *Prunus verrucosa*

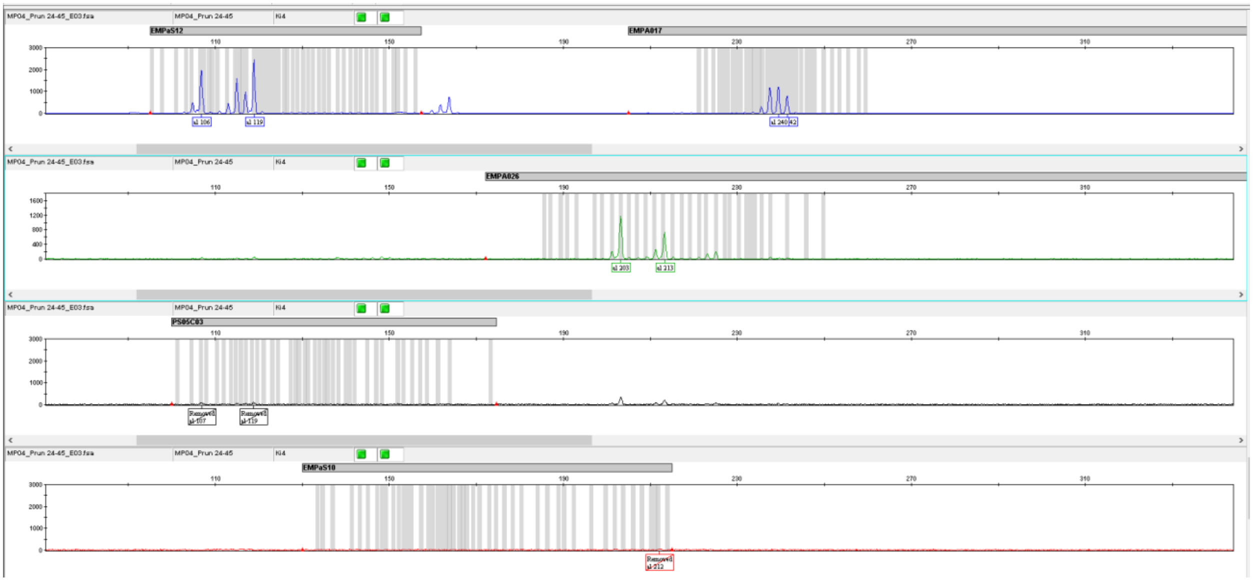

Primer Mix 03. Accessions: Prun24_045. *Prunus verrucosa*
